# 4-Imino-2,7-dimethyl-5,6,7,8-tetra­hydro-4*H*-1-benzothieno[2,3-*d*]pyrimidin-3-amine

**DOI:** 10.1107/S1600536812031893

**Published:** 2012-07-18

**Authors:** Mallikarjun B. Kalashetti, Nikhath Fathima, Ashraf Y. Khan, Noor Shahina Begum, I. M. Khazi

**Affiliations:** a Department of Chemistry, Karnatak University, Dharwad 580 003, India; b Department of Studies in Chemistry, Bangalore University, Bangalore 560 001, India

## Abstract

In the title compound, C_12_H_16_N_4_S, the fused benzothio­phene and the pyrimidine rings are coplanar [dihedral angle = 1.61 (6)°]. Three C atoms of the cyclohexene ring (at positions 3, 6 and 7) are disordered over two sites with an occupancy ratio of 0.702 (8):0.298 (8). The cyclo­hexene ring in both the major and minor components adopts a half-chair conformation. The crystal structure is stabilized by N—H⋯N and C—H⋯N inter­actions, resulting in the formation of inversion dimers with *R*_2_^2^(10) and *R*_2_^2^(12) graph-set motifs.

## Related literature

For the biological activity of thio­phenes, benzothio­phenes and pyrimidines, see: Pathak *et al.* (1991 ▶); Shishoo & Jain (1992 ▶). For a related crystal structure, see: Panchamukhi *et al.* (2011 ▶). For graph-set notations, see: Bernstein *et al.* (1995 ▶).
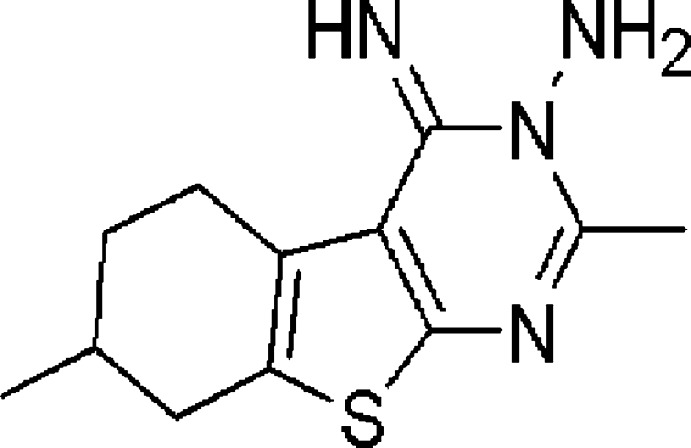


## Experimental

### 

#### Crystal data

C_12_H_16_N_4_S
*M* *_r_* = 248.35
Triclinic, 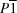

*a* = 6.7514 (5) Å
*b* = 8.7139 (6) Å
*c* = 11.8309 (9) Å
α = 97.221 (4)°
β = 102.820 (4)°
γ = 112.482 (3)°
*V* = 609.73 (8) Å^3^
*Z* = 2
Mo *K*α radiation
μ = 0.25 mm^−1^
*T* = 296 K
0.18 × 0.16 × 0.16 mm

#### Data collection

Bruker SMART APEX CCD detector diffractometer
Absorption correction: multi-scan (*SADABS*; Bruker, 1998 ▶) *T*_min_ = 0.957, *T*_max_ = 0.961
11883 measured reflections
2641 independent reflections
2378 reflections with *I* > 2σ(*I*)
*R*_int_ = 0.018

#### Refinement

*R*[*F* ^2^ > 2σ(*F* ^2^)] = 0.038
*wR*(*F* ^2^) = 0.112
*S* = 1.08
2641 reflections
184 parameters
H-atom parameters constrained
Δρ_max_ = 0.23 e Å^−3^
Δρ_min_ = −0.31 e Å^−3^

### 

Data collection: *SMART* (Bruker, 1998 ▶); cell refinement: *SAINT-Plus* (Bruker, 1998 ▶); data reduction: *SAINT-Plus*; program(s) used to solve structure: *SHELXS97* (Sheldrick, 2008 ▶); program(s) used to refine structure: *SHELXL97* (Sheldrick, 2008 ▶); molecular graphics: *ORTEP-3* (Farrugia, 1997 ▶) and *CAMERON* (Watkin *et al.*, 1996 ▶); software used to prepare material for publication: *WinGX* (Farrugia, 1999 ▶).

## Supplementary Material

Crystal structure: contains datablock(s) global, I. DOI: 10.1107/S1600536812031893/pv2567sup1.cif

Structure factors: contains datablock(s) I. DOI: 10.1107/S1600536812031893/pv2567Isup2.hkl

Supplementary material file. DOI: 10.1107/S1600536812031893/pv2567Isup3.cml

Additional supplementary materials:  crystallographic information; 3D view; checkCIF report

## Figures and Tables

**Table 1:** Hydrogen-bond geometry (Å, °)

*D*—H⋯*A*	*D*—H	H⋯*A*	*D*⋯*A*	*D*—H⋯*A*
N3—H3*B*⋯N4^i^	0.89	2.40	3.117 (2)	137
C5—H5*B*⋯N4^ii^	0.96	2.67	3.587 (2)	160

Symmetry codes: (i) 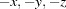; (ii) 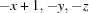.

**Fig. 1.**
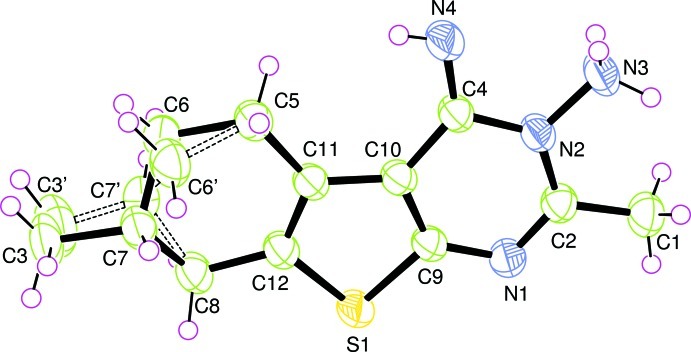
The molecular structure of the title compound with the atom numbering scheme. Displacement ellipsoids are drawn at the 50% probability level. H atoms are presented as small spheres of arbitrary radius. C6 and C7 are disordered over sites C6/C6' and C7/C7', respectively. The H-atoms of the NH2 group are disordered over three sites with s.o.f 0.667.

**Fig. 2.**
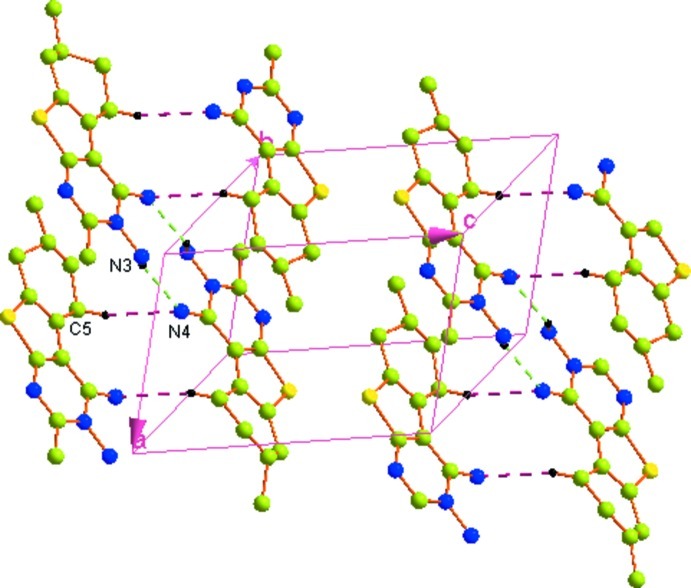
A view of the intermolecular hydrogen bonds(dotted lines) in the crystal structure of the title compound. H atoms non participating in H-bonding were ommitted for clarity.

## supplementary crystallographic information

### Comment

Thienopyrimidine derivatives are reported to have a wide range of biological and medicinal applications (Pathak *et al.*, 1991). The chemistry of thieophenes and benzothiophenes is well documented in the literature as they possess wide spectrum of biological activities (Shishoo & Jain, 1992).

In the title compound the pyrimidine ring is substituted with the benzothiophene moiety at one end and the methyl and imino groups at the other end. The carbon atoms C6 and C7 are disordered over two sites (C6/C6' and C7/C7') with site occupancy factors 0.7022 (6) and 0.2071 (5) resulting in a major and a minor conformers. The H-atoms of the NH2 group are also disordered over three sites with each H-atom having a site occupancy factor of 0.6667. The cyclohexene ring in both the conformers is in the half chair conformation with C6 and C7 atoms being deviated from the rest of the ring atoms by 0.376 (3) and -0.345 (2)° A for the major conformer. The C6' and C7' atoms are deviated by -0.515 (8) and -0.409 (7)° A for the minor conformer respectively. The fused benzothiophene and the pyrimidine ring are coplanar with the dihedral angle 1.609 (6)°. The N(2) atom of the pyrimidine ring is in the planar trigonal configuration. The crystal structure is stabilized by intermolecular N—H···N and C—H···N interactions resulting in centrosymmetric head-to-head dimers (Fig. 2 and Tab. 1) corresponding to the graph set of *R*^2^_2_(10) and *R*^2^_2_(12) motifs (Bernstein *et al.*, 1995). The bond distances and angles in the title compound agree very well with the corresponding bond distances and angles reported in a closely related compound (Panchamukhi *et al.*, 2011).

### Experimental

A mixture of *N*-(3-cyano-6-methyl-4,5,6,7-tetrahydro-benzo[*b*] thiophen-2-yl)-acetimidic acid ethyl ester (1.5 g 5.7 mmol) and hydrazine hydrate (10 ml) was stirred at room temperature for 3 h. The solid separated was filtered, washed with water and recrystallized from ethanol to yield crystals of the title compound suitable for X-ray crystallographic analysis; yield 1.12 g (79%), melting point 430–431 K.

### Refinement

The H atoms were placed at calculated positions in the riding model approximation with N—H = 0.89 and 0.75° A for amine and imine H-atoms, respectively, and C—H = 0.96, 0.97 and 0.98 Å for methyl, methylene and methyne H-atoms, respectively, with *U*_iso_(H) = 1.5*U*_eq_(methyl C) and 1.2*U*_eq_(non-methyl C/N).

### Crystal data

**Table:**

C_12_H_16_N_4_S	*Z* = 2
*M**_r_* = 248.35	*F*(000) = 264
Triclinic, *P*1	*D*_x_ = 1.353 Mg m^−^^3^
Hall symbol: -P 1	Mo *K*α radiation, λ = 0.71073 Å
*a* = 6.7514 (5) Å	Cell parameters from 2641 reflections
*b* = 8.7139 (6) Å	θ = 1.8–27.0°
*c* = 11.8309 (9) Å	µ = 0.25 mm^−^^1^
α = 97.221 (4)°	*T* = 296 K
β = 102.820 (4)°	Block, yellow
γ = 112.482 (3)°	0.18 × 0.16 × 0.16 mm
*V* = 609.73 (8) Å^3^	

### Data collection

**Table:**

Bruker SMART APEX CCD detector diffractometer	2641 independent reflections
Radiation source: fine-focus sealed tube	2378 reflections with *I* > 2σ(*I*)
Graphite monochromator	*R*_int_ = 0.018
ω scans	θ_max_ = 27.0°, θ_min_ = 1.8°
Absorption correction: multi-scan (*SADABS*; Bruker, 1998)	*h* = −8→8
*T*_min_ = 0.957, *T*_max_ = 0.961	*k* = −11→11
11883 measured reflections	*l* = −15→15

### Refinement

**Table:**

Refinement on *F*^2^	Primary atom site location: structure-invariant direct methods
Least-squares matrix: full	Secondary atom site location: difference Fourier map
*R*[*F*^2^ > 2σ(*F*^2^)] = 0.038	Hydrogen site location: inferred from neighbouring sites
*wR*(*F*^2^) = 0.112	H-atom parameters constrained
*S* = 1.08	*w* = 1/[σ^2^(*F*_o_^2^) + (0.0684*P*)^2^ + 0.0963*P*] where *P* = (*F*_o_^2^ + 2*F*_c_^2^)/3
2641 reflections	(Δ/σ)_max_ < 0.001
184 parameters	Δρ_max_ = 0.23 e Å^−^^3^
0 restraints	Δρ_min_ = −0.31 e Å^−^^3^

### Special details

**Table:**

Geometry. All s.u.'s (except the s.u. in the dihedral angle between two l.s. planes) are estimated using the full covariance matrix. The cell s.u.'s are taken into account individually in the estimation of s.u.'s in distances, angles and torsion angles; correlations between s.u.'s in cell parameters are only used when they are defined by crystal symmetry. An approximate (isotropic) treatment of cell s.u.'s is used for estimating s.u.'s involving l.s. planes.
Refinement. Refinement of *F*^2^ against ALL reflections. The weighted *R*-factor *wR* and goodness of fit *S* are based on *F*^2^, conventional *R*-factors *R* are based on *F*, with *F* set to zero for negative *F*^2^. The threshold expression of *F*^2^ > 2σ(*F*^2^) is used only for calculating *R*-factors(gt) *etc*. and is not relevant to the choice of reflections for refinement. *R*-factors based on *F*^2^ are statistically about twice as large as those based on *F*, and *R*- factors based on ALL data will be even larger.

### Fractional atomic coordinates and isotropic or equivalent isotropic displacement parameters (Å^2^)

**Table:**

	*x*	*y*	*z*	*U*_iso_*/*U*_eq_	Occ. (<1)
S1	1.01003 (6)	0.58317 (4)	0.33947 (3)	0.04948 (15)	
N1	0.6333 (2)	0.59256 (15)	0.20955 (11)	0.0467 (3)	
N2	0.33573 (19)	0.33614 (14)	0.09087 (10)	0.0423 (3)	
N3	0.1200 (2)	0.26296 (17)	0.00579 (13)	0.0562 (4)	
H3A	0.0589	0.3371	0.0081	0.084*	0.667
H3B	0.0330	0.1680	0.0228	0.084*	0.667
H3C	0.1328	0.2380	−0.0667	0.084*	0.667
N4	0.3265 (2)	0.06821 (16)	0.06949 (12)	0.0537 (3)	
H4	0.3922	0.0196	0.0911	0.064*	
C1	0.2976 (3)	0.6054 (2)	0.09622 (16)	0.0554 (4)	
H1A	0.3829	0.7246	0.1342	0.083*	
H1B	0.1612	0.5628	0.1181	0.083*	
H1C	0.2624	0.5908	0.0113	0.083*	
C2	0.4318 (2)	0.50924 (17)	0.13533 (13)	0.0424 (3)	
C4	0.4389 (2)	0.22735 (17)	0.12130 (12)	0.0396 (3)	
C5	0.7837 (3)	0.07178 (18)	0.24560 (14)	0.0497 (4)	
H5A	0.6800	0.0160	0.2872	0.060*	
H5B	0.7192	0.0146	0.1627	0.060*	
C3	1.3585 (14)	0.1498 (15)	0.4674 (10)	0.0689 (15)	0.702 (8)
H3D	1.3159	0.0309	0.4658	0.103*	0.702 (8)
H3E	1.4308	0.2156	0.5482	0.103*	0.702 (8)
H3F	1.4600	0.1856	0.4206	0.103*	0.702 (8)
C6	1.0074 (5)	0.0565 (3)	0.2941 (3)	0.0509 (8)	0.702 (8)
H6A	0.9744	−0.0599	0.3010	0.061*	0.702 (8)
H6B	1.0937	0.0813	0.2379	0.061*	0.702 (8)
C7	1.1470 (4)	0.1779 (3)	0.4152 (3)	0.0477 (8)	0.702 (8)
H7A	1.0552	0.1574	0.4701	0.057*	0.702 (8)
C3'	1.355 (4)	0.150 (4)	0.435 (2)	0.093 (8)	0.298 (8)
H3AA	1.5012	0.2346	0.4410	0.140*	0.298 (8)
H3AB	1.3436	0.0386	0.4057	0.140*	0.298 (8)
H3AC	1.3323	0.1576	0.5127	0.140*	0.298 (8)
C6'	0.9513 (13)	0.0591 (8)	0.3484 (8)	0.057 (2)	0.298 (8)
H6AA	0.9204	0.0873	0.4226	0.068*	0.298 (8)
H6AB	0.9393	−0.0567	0.3371	0.068*	0.298 (8)
C7'	1.1840 (11)	0.1812 (8)	0.3536 (8)	0.0525 (18)	0.298 (8)
H7AA	1.2031	0.1697	0.2737	0.063*	0.298 (8)
C8	1.2128 (2)	0.3636 (2)	0.40058 (14)	0.0492 (3)	
H8A	1.3251	0.3925	0.3595	0.059*	
H8B	1.2756	0.4402	0.4780	0.059*	
C9	0.7411 (2)	0.49320 (17)	0.24249 (12)	0.0405 (3)	
C10	0.6592 (2)	0.31806 (16)	0.20678 (11)	0.0384 (3)	
C11	0.8178 (2)	0.25520 (17)	0.25982 (12)	0.0392 (3)	
C12	1.0128 (2)	0.38438 (18)	0.33207 (12)	0.0422 (3)	

### Atomic displacement parameters (Å^2^)

**Table:**

	*U*^11^	*U*^22^	*U*^33^	*U*^12^	*U*^13^	*U*^23^
S1	0.0383 (2)	0.0365 (2)	0.0568 (2)	0.01117 (15)	−0.00373 (15)	0.00092 (15)
N1	0.0416 (6)	0.0355 (6)	0.0558 (7)	0.0156 (5)	0.0042 (5)	0.0066 (5)
N2	0.0323 (6)	0.0381 (6)	0.0484 (6)	0.0129 (5)	0.0015 (5)	0.0078 (5)
N3	0.0367 (7)	0.0479 (7)	0.0693 (8)	0.0171 (6)	−0.0077 (6)	0.0087 (6)
N4	0.0418 (7)	0.0370 (6)	0.0655 (8)	0.0139 (5)	−0.0062 (6)	0.0039 (5)
C1	0.0487 (9)	0.0457 (8)	0.0701 (10)	0.0244 (7)	0.0065 (7)	0.0138 (7)
C2	0.0394 (7)	0.0385 (7)	0.0481 (7)	0.0165 (6)	0.0100 (6)	0.0113 (6)
C4	0.0341 (6)	0.0369 (6)	0.0422 (6)	0.0131 (5)	0.0046 (5)	0.0085 (5)
C5	0.0458 (8)	0.0382 (7)	0.0546 (8)	0.0165 (6)	−0.0012 (6)	0.0078 (6)
C3	0.045 (2)	0.067 (3)	0.088 (4)	0.0301 (18)	−0.0069 (19)	0.022 (2)
C6	0.0469 (13)	0.0476 (12)	0.0571 (18)	0.0251 (11)	0.0064 (11)	0.0077 (11)
C7	0.0400 (12)	0.0492 (12)	0.0517 (17)	0.0210 (9)	0.0043 (10)	0.0140 (11)
C3'	0.078 (8)	0.068 (7)	0.115 (17)	0.028 (6)	−0.012 (8)	0.037 (9)
C6'	0.058 (4)	0.053 (3)	0.058 (4)	0.026 (3)	0.006 (3)	0.021 (3)
C7'	0.051 (3)	0.057 (3)	0.055 (4)	0.031 (3)	0.010 (3)	0.016 (3)
C8	0.0351 (7)	0.0485 (8)	0.0543 (8)	0.0154 (6)	0.0010 (6)	0.0085 (6)
C9	0.0356 (7)	0.0367 (6)	0.0418 (6)	0.0129 (5)	0.0041 (5)	0.0056 (5)
C10	0.0352 (7)	0.0359 (7)	0.0383 (6)	0.0127 (5)	0.0053 (5)	0.0071 (5)
C11	0.0365 (6)	0.0384 (7)	0.0380 (6)	0.0147 (5)	0.0051 (5)	0.0076 (5)
C12	0.0358 (7)	0.0413 (7)	0.0436 (7)	0.0149 (6)	0.0046 (5)	0.0077 (5)

### Geometric parameters (Å, º)

**Table:**

S1—C9	1.7288 (14)	C3—H3D	0.9600
S1—C12	1.7308 (14)	C3—H3E	0.9600
N1—C2	1.3051 (18)	C3—H3F	0.9600
N1—C9	1.3682 (18)	C6—C7	1.523 (5)
N2—C2	1.3688 (18)	C6—H6A	0.9700
N2—C4	1.4118 (17)	C6—H6B	0.9700
N2—N3	1.4211 (16)	C7—C8	1.549 (3)
N3—H3A	0.8900	C7—H7A	0.9800
N3—H3B	0.8900	C3'—C7'	1.47 (2)
N3—H3C	0.8900	C3'—H3AA	0.9600
N4—C4	1.2844 (18)	C3'—H3AB	0.9600
N4—H4	0.7500	C3'—H3AC	0.9600
C1—C2	1.4933 (19)	C6'—C7'	1.506 (13)
C1—H1A	0.9600	C6'—H6AA	0.9700
C1—H1B	0.9600	C6'—H6AB	0.9700
C1—H1C	0.9600	C7'—C8	1.538 (6)
C4—C10	1.4465 (17)	C7'—H7AA	0.9800
C5—C11	1.5067 (19)	C8—C12	1.5008 (19)
C5—C6'	1.513 (6)	C8—H8A	0.9599
C5—C6	1.554 (3)	C8—H8B	0.9600
C5—H5A	0.9600	C9—C10	1.3793 (19)
C5—H5B	0.9601	C10—C11	1.4410 (18)
C3—C7	1.548 (8)	C11—C12	1.3624 (19)
			
C9—S1—C12	91.18 (6)	C6—C7—H7A	108.7
C2—N1—C9	114.93 (12)	C3—C7—H7A	108.7
C2—N2—C4	124.49 (12)	C8—C7—H7A	108.7
C2—N2—N3	117.21 (11)	C7'—C3'—H3AA	109.5
C4—N2—N3	118.27 (11)	C7'—C3'—H3AB	109.5
N2—N3—H3A	109.5	H3AA—C3'—H3AB	109.5
N2—N3—H3B	109.5	C7'—C3'—H3AC	109.5
H3A—N3—H3B	109.5	H3AA—C3'—H3AC	109.5
N2—N3—H3C	109.5	H3AB—C3'—H3AC	109.5
H3A—N3—H3C	109.5	C7'—C6'—C5	109.0 (7)
H3B—N3—H3C	109.5	C7'—C6'—H6AA	109.9
C4—N4—H4	109.5	C5—C6'—H6AA	109.9
C2—C1—H1A	109.5	C7'—C6'—H6AB	109.9
C2—C1—H1B	109.5	C5—C6'—H6AB	109.9
H1A—C1—H1B	109.5	H6AA—C6'—H6AB	108.3
C2—C1—H1C	109.5	C3'—C7'—C6'	111.2 (13)
H1A—C1—H1C	109.5	C3'—C7'—C8	108.3 (13)
H1B—C1—H1C	109.5	C6'—C7'—C8	108.0 (7)
N1—C2—N2	123.07 (13)	C3'—C7'—H7AA	109.8
N1—C2—C1	119.13 (13)	C6'—C7'—H7AA	109.8
N2—C2—C1	117.80 (13)	C8—C7'—H7AA	109.8
N4—C4—N2	116.33 (12)	C12—C8—C7'	108.5 (2)
N4—C4—C10	130.93 (12)	C12—C8—C7	111.03 (14)
N2—C4—C10	112.74 (11)	C12—C8—H8A	109.5
C11—C5—C6'	108.9 (3)	C7'—C8—H8A	82.4
C11—C5—C6	112.04 (14)	C7—C8—H8A	109.6
C11—C5—H5A	109.1	C12—C8—H8B	109.5
C6'—C5—H5A	82.6	C7'—C8—H8B	133.9
C6—C5—H5A	109.5	C7—C8—H8B	109.2
C11—C5—H5B	109.2	H8A—C8—H8B	108.1
C6'—C5—H5B	133.9	N1—C9—C10	127.20 (13)
C6—C5—H5B	109.0	N1—C9—S1	120.97 (10)
H5A—C5—H5B	108.0	C10—C9—S1	111.83 (10)
C7—C6—C5	112.0 (3)	C9—C10—C11	112.38 (12)
C7—C6—H6A	109.2	C9—C10—C4	117.49 (12)
C5—C6—H6A	109.2	C11—C10—C4	130.08 (12)
C7—C6—H6B	109.2	C12—C11—C10	111.76 (12)
C5—C6—H6B	109.2	C12—C11—C5	120.89 (13)
H6A—C6—H6B	107.9	C10—C11—C5	127.32 (12)
C6—C7—C3	111.4 (5)	C11—C12—C8	125.54 (13)
C6—C7—C8	108.5 (3)	C11—C12—S1	112.85 (11)
C3—C7—C8	110.7 (5)	C8—C12—S1	121.60 (11)
			
C9—N1—C2—N2	−1.5 (2)	C12—S1—C9—N1	179.87 (12)
C9—N1—C2—C1	178.47 (13)	C12—S1—C9—C10	0.01 (11)
C4—N2—C2—N1	1.5 (2)	N1—C9—C10—C11	−179.52 (13)
N3—N2—C2—N1	−176.67 (13)	S1—C9—C10—C11	0.34 (15)
C4—N2—C2—C1	−178.53 (13)	N1—C9—C10—C4	2.7 (2)
N3—N2—C2—C1	3.3 (2)	S1—C9—C10—C4	−177.40 (10)
C2—N2—C4—N4	−178.75 (14)	N4—C4—C10—C9	176.81 (15)
N3—N2—C4—N4	−0.62 (19)	N2—C4—C10—C9	−2.54 (18)
C2—N2—C4—C10	0.71 (19)	N4—C4—C10—C11	−0.5 (3)
N3—N2—C4—C10	178.83 (12)	N2—C4—C10—C11	−179.81 (13)
C11—C5—C6—C7	45.4 (4)	C9—C10—C11—C12	−0.62 (17)
C6'—C5—C6—C7	−45.0 (5)	C4—C10—C11—C12	176.76 (13)
C5—C6—C7—C3	174.9 (5)	C9—C10—C11—C5	177.34 (13)
C5—C6—C7—C8	−63.1 (4)	C4—C10—C11—C5	−5.3 (2)
C11—C5—C6'—C7'	−55.1 (9)	C6'—C5—C11—C12	18.5 (5)
C6—C5—C6'—C7'	46.4 (7)	C6—C5—C11—C12	−14.3 (3)
C5—C6'—C7'—C3'	−168.6 (14)	C6'—C5—C11—C10	−159.3 (5)
C5—C6'—C7'—C8	72.8 (10)	C6—C5—C11—C10	167.9 (2)
C3'—C7'—C8—C12	−169.0 (12)	C10—C11—C12—C8	−179.79 (13)
C6'—C7'—C8—C12	−48.4 (8)	C5—C11—C12—C8	2.1 (2)
C3'—C7'—C8—C7	−68.8 (13)	C10—C11—C12—S1	0.63 (16)
C6'—C7'—C8—C7	51.8 (7)	C5—C11—C12—S1	−177.49 (11)
C6—C7—C8—C12	48.3 (3)	C7'—C8—C12—C11	13.1 (5)
C3—C7—C8—C12	170.8 (5)	C7—C8—C12—C11	−19.6 (3)
C6—C7—C8—C7'	−42.9 (5)	C7'—C8—C12—S1	−167.3 (4)
C3—C7—C8—C7'	79.6 (7)	C7—C8—C12—S1	159.94 (19)
C2—N1—C9—C10	−0.6 (2)	C9—S1—C12—C11	−0.37 (11)
C2—N1—C9—S1	179.55 (10)	C9—S1—C12—C8	−179.97 (13)

### Hydrogen-bond geometry (Å, º)

**Table:**

*D*—H···*A*	*D*—H	H···*A*	*D*···*A*	*D*—H···*A*
N3—H3*B*···N4^i^	0.89	2.40	3.117 (2)	137
C5—H5*B*···N4^ii^	0.96	2.67	3.587 (2)	160

Symmetry codes: (i) −*x*, −*y*, −*z*; (ii) −*x*+1, −*y*, −*z*.
